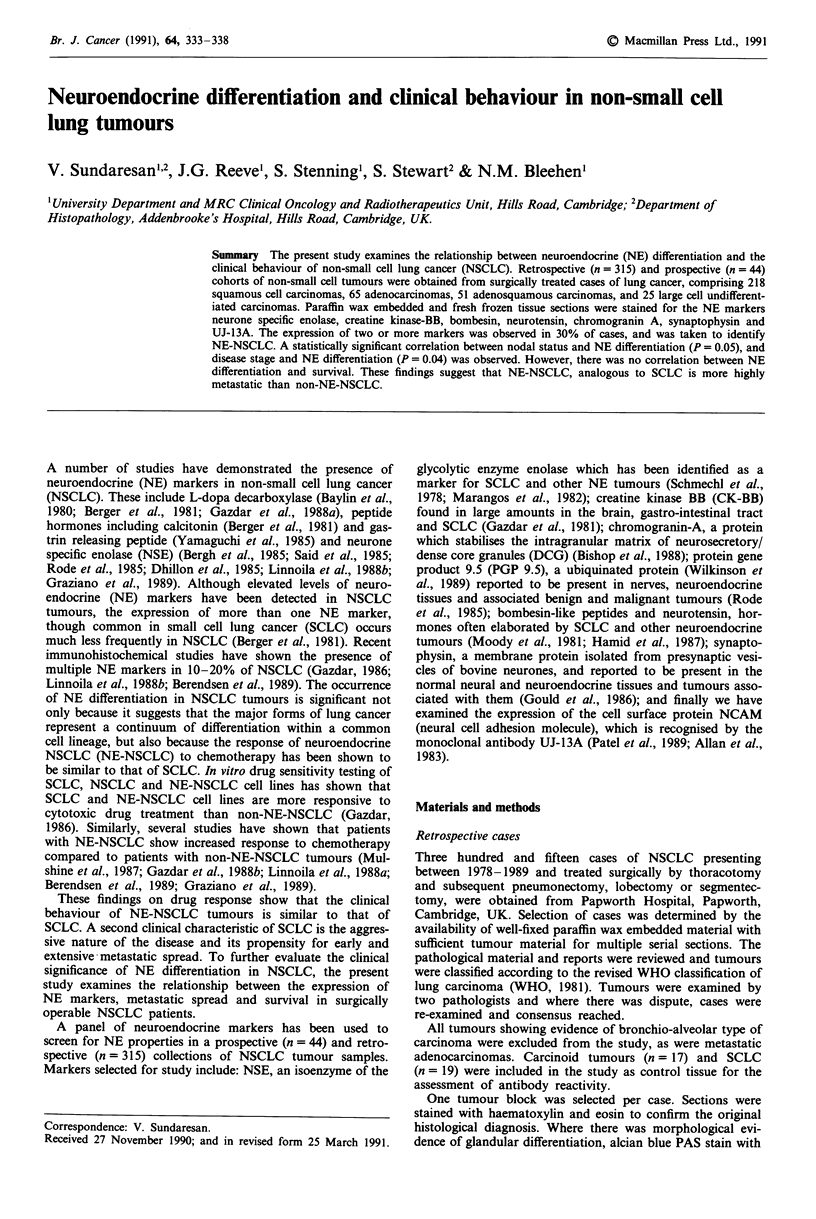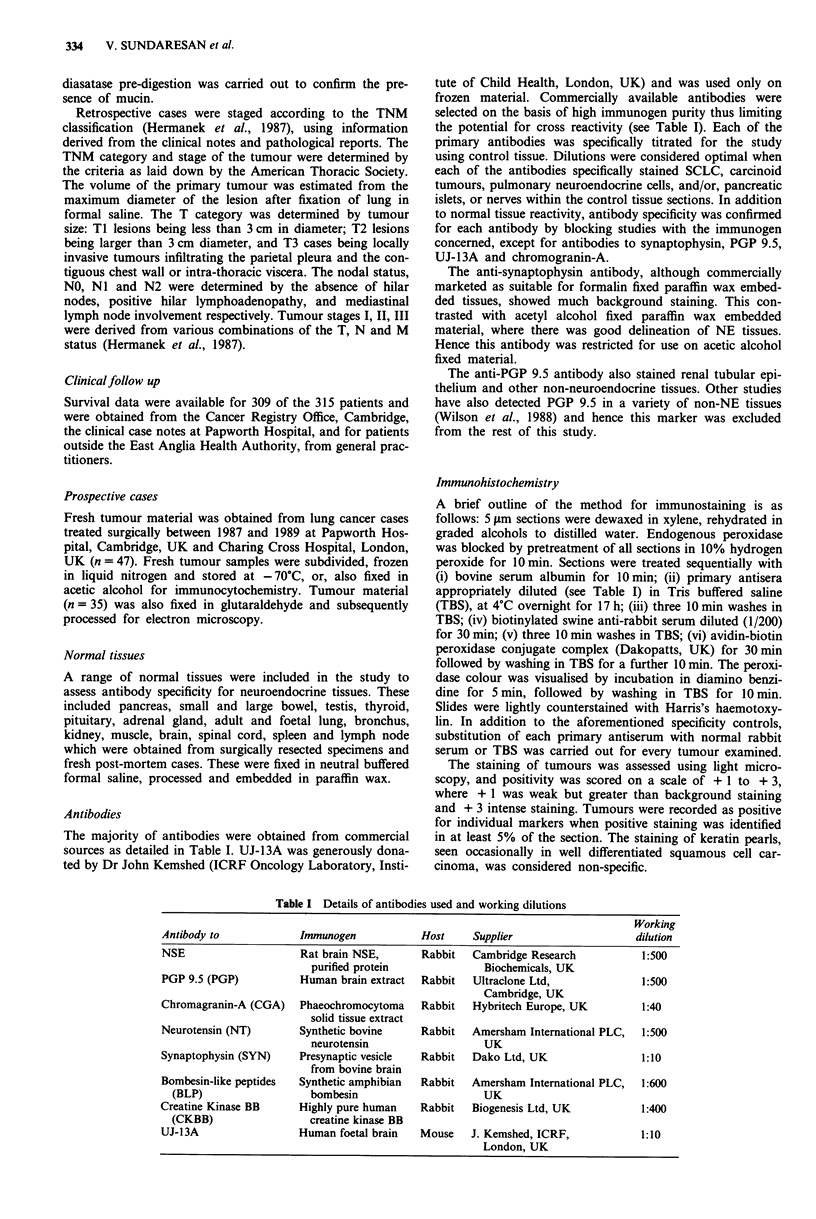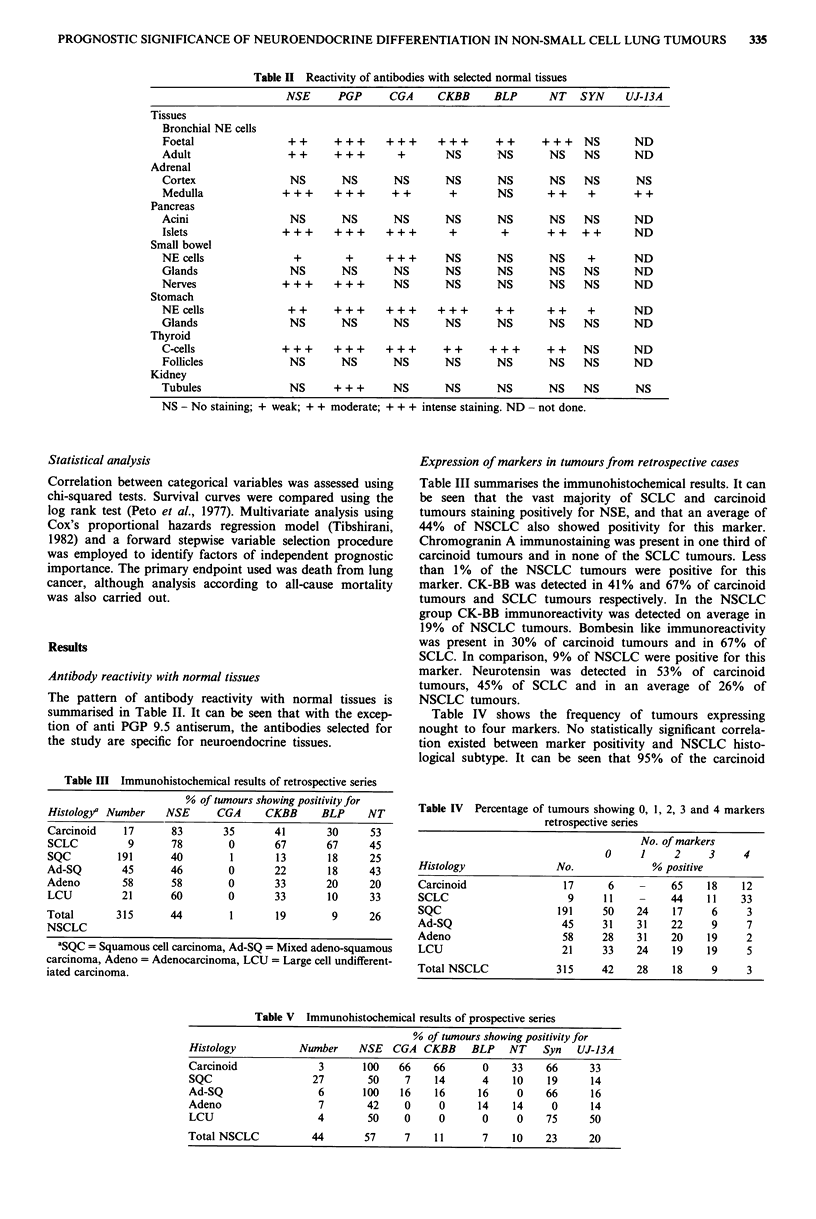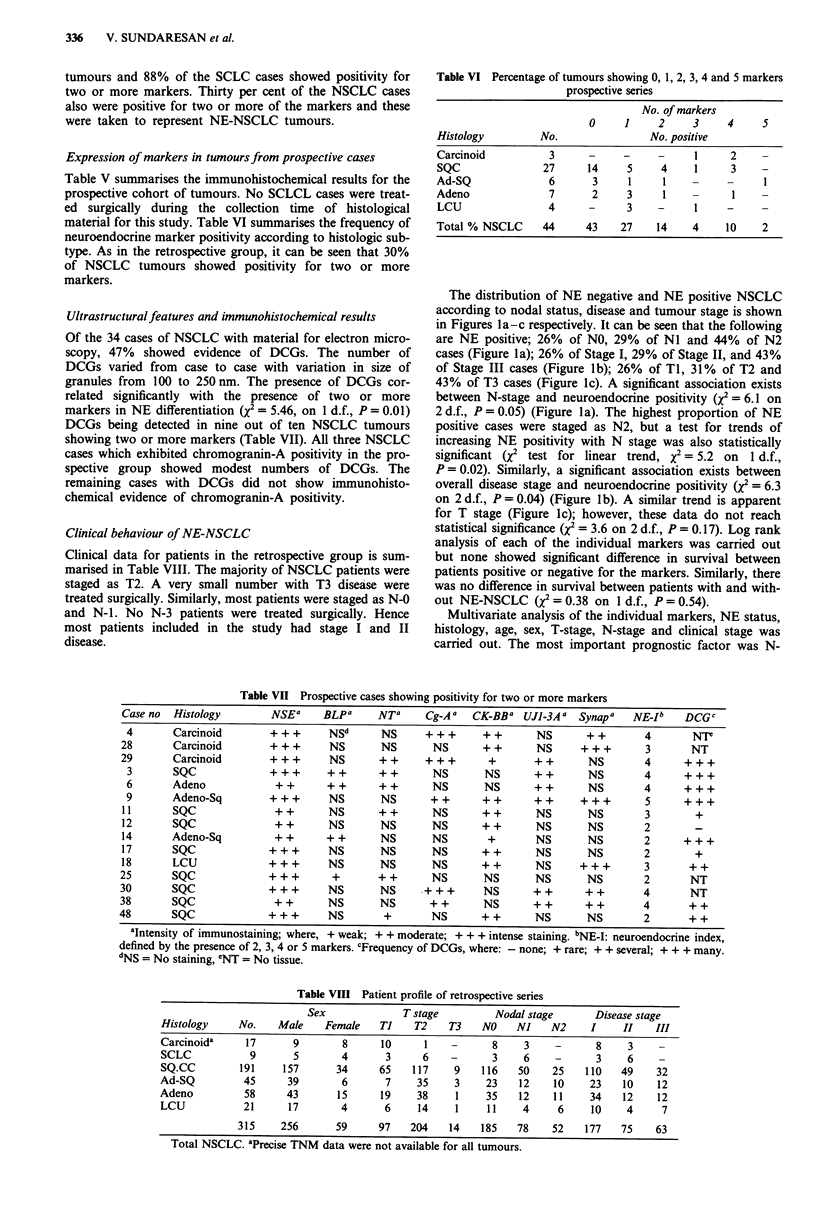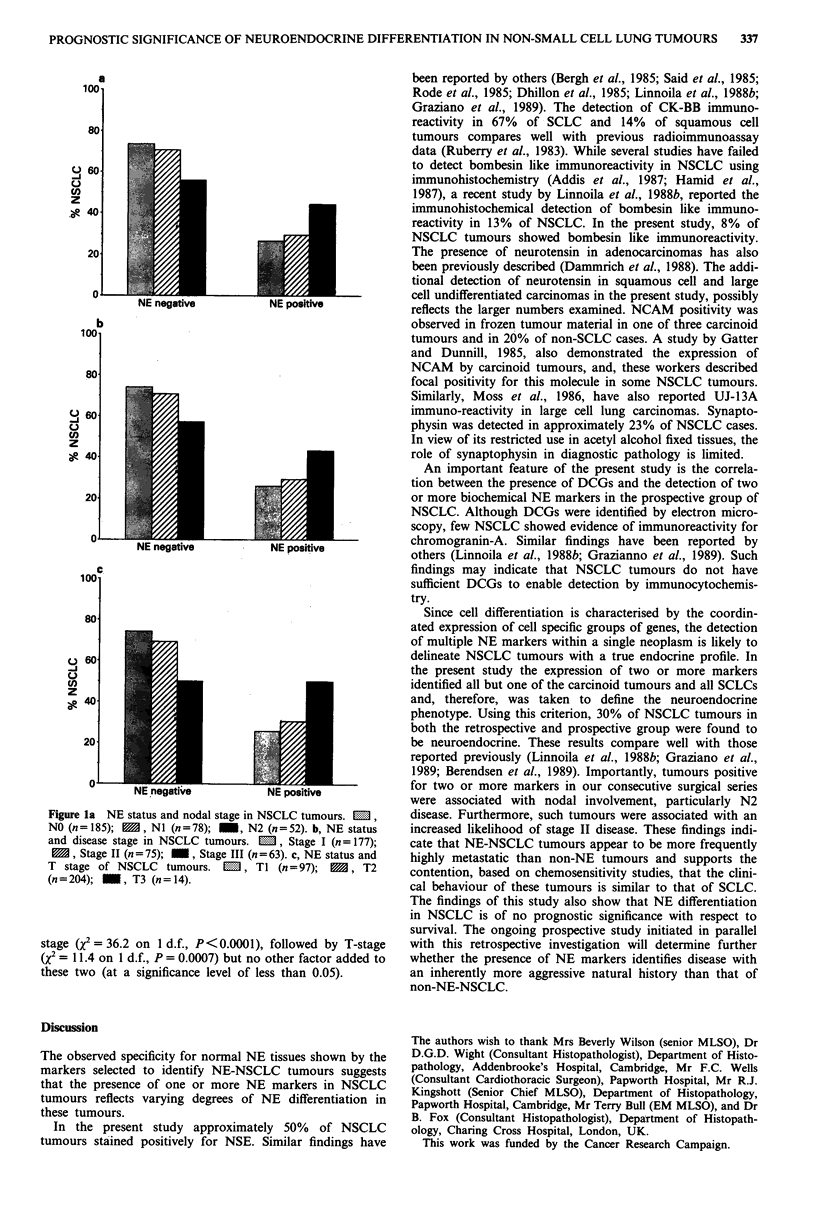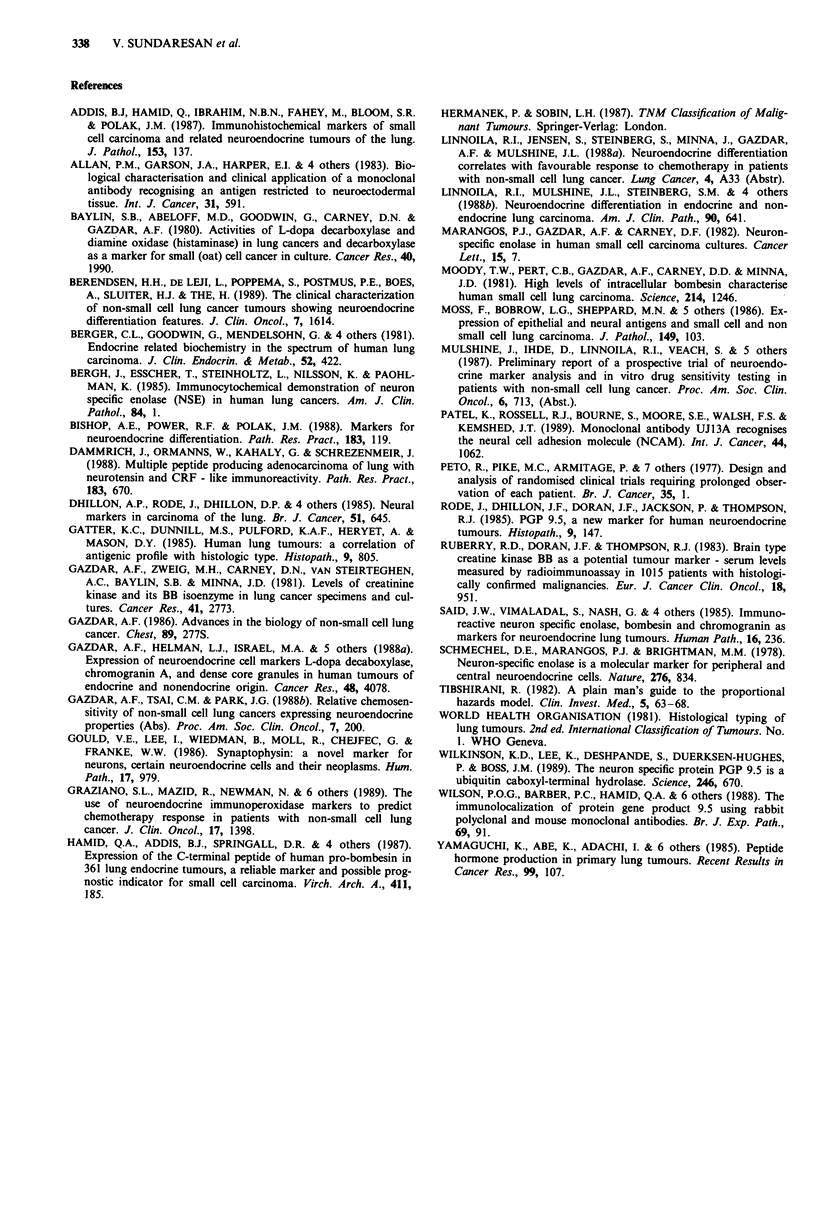# Neuroendocrine differentiation and clinical behaviour in non-small cell lung tumours.

**DOI:** 10.1038/bjc.1991.301

**Published:** 1991-08

**Authors:** V. Sundaresan, J. G. Reeve, S. Stenning, S. Stewart, N. M. Bleehen

**Affiliations:** University Department, Cambridge, UK.

## Abstract

The present study examines the relationship between neuroendocrine (NE) differentiation and the clinical behaviour of non-small cell lung cancer (NSCLC). Retrospective (n = 315) and prospective (n = 44) cohorts of non-small cell tumours were obtained from surgically treated cases of lung cancer, comprising 218 squamous cell carcinomas, 65 adenocarcinomas, 51 adenosquamous carcinomas, and 25 large cell undifferentiated carcinomas. Paraffin wax embedded and fresh frozen tissue sections were stained for the NE markers neurone specific enolase, creatine kinase-BB, bombesin, neurotensin, chromogranin A, synaptophysin and UJ-13A. The expression of two or more markers was observed in 30% of cases, and was taken to identify NE-NSCLC. A statistically significant correlation between nodal status and NE differentiation (P = 0.05), and disease stage and NE differentiation (P = 0.04) was observed. However, there was no correlation between NE differentiation and survival. These findings suggest that NE-NSCLC, analogous to SCLC is more highly metastatic than non-NE-NSCLC.


*Br. J. Cancer* (1991), **64**, 333–338 © Macmillan Press Ltd., 1991

### Neuroendocrine differentiation and clinical behaviour in non-small cell lung tumours

V. Sundaresan[1,2], J.G. Reeve[1], S. Stenning[1], S. Stewart[2] & N.M. Bleehen[1]

[1]*University Department and MRC Clinical Oncology and Radiotherapeutics Unit, Hills Road, Cambridge;* [2]*Department of Histopathology, Addenbrooke's Hospital, Hills Road, Cambridge, UK.*

**Summary** The present study examines the relationship between neuroendocrine (NE) differentiation and the clinical behaviour of non-small cell lung cancer (NSCLC). Retrospective ($n = 315$) and prospective ($n = 44$) cohorts of non-small cell tumours were obtained from surgically treated cases of lung cancer, comprising 218 squamous cell carcinomas, 65 adenocarcinomas, 51 adenosquamous carcinomas, and 25 large cell undifferentiated carcinomas. Paraffin wax embedded and fresh frozen tissue sections were stained for the NE markers neurone specific enolase, creatine kinase-BB, bombesin, neurotensin, chromogranin A, synaptophysin and UJ-13A. The expression of two or more markers was observed in 30% of cases, and was taken to identify NE-NSCLC. A statistically significant correlation between nodal status and NE differentiation ($P = 0.05$), and disease stage and NE differentiation ($P = 0.04$) was observed. However, there was no correlation between NE differentiation and survival. These findings suggest that NE-NSCLC, analogous to SCLC is more highly metastatic than non-NE-NSCLC.

A number of studies have demonstrated the presence of neuroendocrine (NE) markers in non-small cell lung cancer (NSCLC). These include L-dopa decarboxylase (Baylin *et al.*, 1980; Berger *et al.*, 1981; Gazdar *et al.*, 1988*a*), peptide hormones including calcitonin (Berger *et al.*, 1981) and gastrin releasing peptide (Yamaguchi *et al.*, 1985) and neurone specific enolase (NSE) (Bergh *et al.*, 1985; Said *et al.*, 1985; Rode *et al.*, 1985; Dhillon *et al.*, 1985; Linnoila *et al.*, 1988*b*; Graziano *et al.*, 1989). Although elevated levels of neuroendocrine (NE) markers have been detected in NSCLC tumours, the expression of more than one NE marker, though common in small cell lung cancer (SCLC) occurs much less frequently in NSCLC (Berger *et al.*, 1981). Recent immunohistochemical studies have shown the presence of multiple NE markers in 10–20% of NSCLC (Gazdar, 1986; Linnoila *et al.*, 1988*b*; Berendsen *et al.*, 1989). The occurrence of NE differentiation in NSCLC tumours is significant not only because it suggests that the major forms of lung cancer represent a continuum of differentiation within a common cell lineage, but also because the response of neuroendocrine NSCLC (NE-NSCLC) to chemotherapy has been shown to be similar to that of SCLC. *In vitro* drug sensitivity testing of SCLC, NSCLC and NE-NSCLC cell lines has shown that SCLC and NE-NSCLC cell lines are more responsive to cytotoxic drug treatment than non-NE-NSCLC (Gazdar, 1986). Similarly, several studies have shown that patients with NE-NSCLC show increased response to chemotherapy compared to patients with non-NE-NSCLC tumours (Mulshine *et al.*, 1987; Gazdar *et al.*, 1988*b*; Linnoila *et al.*, 1988*a*; Berendsen *et al.*, 1989; Graziano *et al.*, 1989).

These findings on drug response show that the clinical behaviour of NE-NSCLC tumours is similar to that of SCLC. A second clinical characteristic of SCLC is the aggressive nature of the disease and its propensity for early and extensive metastatic spread. To further evaluate the clinical significance of NE differentiation in NSCLC, the present study examines the relationship between the expression of NE markers, metastatic spread and survival in surgically operable NSCLC patients.

A panel of neuroendocrine markers has been used to screen for NE properties in a prospective ($n = 44$) and retrospective ($n = 315$) collections of NSCLC tumour samples. Markers selected for study include: NSE, an isoenzyme of the glycolytic enzyme enolase which has been identified as a marker for SCLC and other NE tumours (Schmechl *et al.*, 1978; Marangos *et al.*, 1982); creatine kinase BB (CK-BB) found in large amounts in the brain, gastro-intestinal tract and SCLC (Gazdar *et al.*, 1981); chromogranin-A, a protein which stabilises the intragranular matrix of neurosecretory/dense core granules (DCG) (Bishop *et al.*, 1988); protein gene product 9.5 (PGP 9.5), a ubiquinated protein (Wilkinson *et al.*, 1989) reported to be present in nerves, neuroendocrine tissues and associated benign and malignant tumours (Rode *et al.*, 1985); bombesin-like peptides and neurotensin, hormones often elaborated by SCLC and other neuroendocrine tumours (Moody *et al.*, 1981; Hamid *et al.*, 1987); synaptophysin, a membrane protein isolated from presynaptic vesicles of bovine neurones, and reported to be present in the normal neural and neuroendocrine tissues and tumours associated with them (Gould *et al.*, 1986); and finally we have examined the expression of the cell surface protein NCAM (neural cell adhesion molecule), which is recognised by the monoclonal antibody UJ-13A (Patel *et al.*, 1989; Allan *et al.*, 1983).

Correspondence: V. Sundaresan.
Received 27 November 1990; and in revised form 25 March 1991.

### Materials and methods

### *Retrospective cases*

Three hundred and fifteen cases of NSCLC presenting between 1978–1989 and treated surgically by thoracotomy and subsequent pneumonectomy, lobectomy or segmentectomy, were obtained from Papworth Hospital, Papworth, Cambridge, UK. Selection of cases was determined by the availability of well-fixed paraffin wax embedded material with sufficient tumour material for multiple serial sections. The pathological material and reports were reviewed and tumours were classified according to the revised WHO classification of lung carcinoma (WHO, 1981). Tumours were examined by two pathologists and where there was dispute, cases were re-examined and consensus reached.

All tumours showing evidence of bronchio-alveolar type of carcinoma were excluded from the study, as were metastatic adenocarcinomas. Carcinoid tumours ($n = 17$) and SCLC ($n = 19$) were included in the study as control tissue for the assessment of antibody reactivity.

One tumour block was selected per case. Sections were stained with haematoxylin and eosin to confirm the original histological diagnosis. Where there was morphological evidence of glandular differentiation, alcian blue PAS stain with diasatase pre-digestion was carried out to confirm the presence of mucin.

Retrospective cases were staged according to the TNM classification (Hermanek et al., 1987), using information derived from the clinical notes and pathological reports. The TNM category and stage of the tumour were determined by the criteria as laid down by the American Thoracic Society. The volume of the primary tumour was estimated from the maximum diameter of the lesion after fixation of lung in formal saline. The T category was determined by tumour size: T1 lesions being less than 3 cm in diameter; T2 lesions being larger than 3 cm diameter, and T3 cases being locally invasive tumours infiltrating the parietal pleura and the contiguous chest wall or intra-thoracic viscera. The nodal status, N0, N1 and N2 were determined by the absence of hilar nodes, positive hilar lymphoadenopathy, and mediastinal lymph node involvement respectively. Tumour stages I, II, III were derived from various combinations of the T, N and M status (Hermanek et al., 1987).

*Clinical follow up*

Survival data were available for 309 of the 315 patients and were obtained from the Cancer Registry Office, Cambridge, the clinical case notes at Papworth Hospital, and for patients outside the East Anglia Health Authority, from general practitioners.

*Prospective cases*

Fresh tumour material was obtained from lung cancer cases treated surgically between 1987 and 1989 at Papworth Hospital, Cambridge, UK and Charing Cross Hospital, London, UK ($n = 47$). Fresh tumour samples were subdivided, frozen in liquid nitrogen and stored at −70°C, or, also fixed in acetic alcohol for immunocytochemistry. Tumour material ($n = 35$) was also fixed in glutaraldehyde and subsequently processed for electron microscopy.

*Normal tissues*

A range of normal tissues were included in the study to assess antibody specificity for neuroendocrine tissues. These included pancreas, small and large bowel, testis, thyroid, pituitary, adrenal gland, adult and foetal lung, bronchus, kidney, muscle, brain, spinal cord, spleen and lymph node which were obtained from surgically resected specimens and fresh post-mortem cases. These were fixed in neutral buffered formal saline, processed and embedded in paraffin wax.

*Antibodies*

The majority of antibodies were obtained from commercial sources as detailed in Table I. UJ-13A was generously donated by Dr John Kemshed (ICRF Oncology Laboratory, Institute of Child Health, London, UK) and was used only on frozen material. Commercially available antibodies were selected on the basis of high immunogen purity thus limiting the potential for cross reactivity (see Table I). Each of the primary antibodies was specifically titrated for the study using control tissue. Dilutions were considered optimal when each of the antibodies specifically stained SCLC, carcinoid tumours, pulmonary neuroendocrine cells, and/or, pancreatic islets, or nerves within the control tissue sections. In addition to normal tissue reactivity, antibody specificity was confirmed for each antibody by blocking studies with the immunogen concerned, except for antibodies to synaptophysin, PGP 9.5, UJ-13A and chromogranin-A.

The anti-synaptophysin antibody, although commercially marketed as suitable for formalin fixed paraffin wax embedded tissues, showed much background staining. This contrasted with acetyl alcohol fixed paraffin wax embedded material, where there was good delineation of NE tissues. Hence this antibody was restricted for use on acetic alcohol fixed material.

The anti-PGP 9.5 antibody also stained renal tubular epithelium and other non-neuroendocrine tissues. Other studies have also detected PGP 9.5 in a variety of non-NE tissues (Wilson et al., 1988) and hence this marker was excluded from the rest of this study.

*Immunohistochemistry*

A brief outline of the method for immunostaining is as follows: 5 μm sections were dewaxed in xylene, rehydrated in graded alcohols to distilled water. Endogenous peroxidase was blocked by pretreatment of all sections in 10% hydrogen peroxide for 10 min. Sections were treated sequentially with (i) bovine serum albumin for 10 min; (ii) primary antisera appropriately diluted (see Table I) in Tris buffered saline (TBS), at 4°C overnight for 17 h; (iii) three 10 min washes in TBS; (iv) biotinylated swine anti-rabbit serum diluted (1/200) for 30 min; (v) three 10 min washes in TBS; (vi) avidin-biotin peroxidase conjugate complex (Dakopatts, UK) for 30 min followed by washing in TBS for a further 10 min. The peroxidase colour was visualised by incubation in diamino benzidine for 5 min, followed by washing in TBS for 10 min. Slides were lightly counterstained with Harris's haemotoxylin. In addition to the aforementioned specificity controls, substitution of each primary antiserum with normal rabbit serum or TBS was carried out for every tumour examined.

The staining of tumours was assessed using light microscopy, and positivity was scored on a scale of + 1 to + 3, where + 1 was weak but greater than background staining and + 3 intense staining. Tumours were recorded as positive for individual markers when positive staining was identified in at least 5% of the section. The staining of keratin pearls, seen occasionally in well differentiated squamous cell carcinoma, was considered non-specific.

**Table I** Details of antibodies used and working dilutions

| *Antibody to* | *Immunogen* | *Host* | *Supplier* | *Working dilution* |
|---|---|---|---|---|
| NSE | Rat brain NSE, purified protein | Rabbit | Cambridge Research Biochemicals, UK | 1:500 |
| PGP 9.5 (PGP) | Human brain extract | Rabbit | Ultraclone Ltd, Cambridge, UK | 1:500 |
| Chromagranin-A (CGA) | Phaeochromocytoma solid tissue extract | Rabbit | Hybritech Europe, UK | 1:40 |
| Neurotensin (NT) | Synthetic bovine neurotensin | Rabbit | Amersham International PLC, UK | 1:500 |
| Synaptophysin (SYN) | Presynaptic vesicle from bovine brain | Rabbit | Dako Ltd, UK | 1:10 |
| Bombesin-like peptides (BLP) | Synthetic amphibian bombesin | Rabbit | Amersham International PLC, UK | 1:600 |
| Creatine Kinase BB (CKBB) | Highly pure human creatine kinase BB | Rabbit | Biogenesis Ltd, UK | 1:400 |
| UJ-13A | Human foetal brain | Mouse | J. Kemshed, ICRF, London, UK | 1:10 |

**Table II** Reactivity of antibodies with selected normal tissues

| | *NSE* | *PGP* | *CGA* | *CKBB* | *BLP* | *NT* | *SYN* | *UJ-13A* |
|---|---|---|---|---|---|---|---|---|
| Tissues | | | | | | | | |
| Bronchial NE cells | | | | | | | | |
| Foetal | + + | + + + | + + + | + + + | + + | + + + | NS | ND |
| Adult | + + | + + + | + | NS | NS | NS | NS | ND |
| Adrenal | | | | | | | | |
| Cortex | NS | NS | NS | NS | NS | NS | NS | NS |
| Medulla | + + + | + + + | + + | + | NS | + + | + | + + |
| Pancreas | | | | | | | | |
| Acini | NS | NS | NS | NS | NS | NS | NS | ND |
| Islets | + + + | + + + | + + + | + | + | + + | + + | ND |
| Small bowel | | | | | | | | |
| NE cells | + | + | + + + | NS | NS | NS | + | ND |
| Glands | NS | NS | NS | NS | NS | NS | NS | ND |
| Nerves | + + + | + + + | NS | NS | NS | NS | NS | ND |
| Stomach | | | | | | | | |
| NE cells | + + | + + + | + + + | + + + | + + | + + | + | ND |
| Glands | NS | NS | NS | NS | NS | NS | NS | ND |
| Thyroid | | | | | | | | |
| C-cells | + + + | + + + | + + + | + + | + + + | + + | NS | ND |
| Follicles | NS | NS | NS | NS | NS | NS | NS | ND |
| Kidney | | | | | | | | |
| Tubules | NS | + + + | NS | NS | NS | NS | NS | NS |

NS – No staining; + weak; + + moderate; + + + intense staining. ND – not done.

*Statistical analysis*

Correlation between categorical variables was assessed using chi-squared tests. Survival curves were compared using the log rank test (Peto *et al.*, 1977). Multivariate analysis using Cox's proportional hazards regression model (Tibshirani, 1982) and a forward stepwise variable selection procedure was employed to identify factors of independent prognostic importance. The primary endpoint used was death from lung cancer, although analysis according to all-cause mortality was also carried out.

### Results

*Antibody reactivity with normal tissues*

The pattern of antibody reactivity with normal tissues is summarised in Table II. It can be seen that with the exception of anti PGP 9.5 antiserum, the antibodies selected for the study are specific for neuroendocrine tissues.

**Table III** Immunohistochemical results of retrospective series

| | | *% of tumours showing positivity for* | | | | |
|---|---|---|---|---|---|---|
| *Histology*[a] | *Number* | *NSE* | *CGA* | *CKBB* | *BLP* | *NT* |
| Carcinoid | 17 | 83 | 35 | 41 | 30 | 53 |
| SCLC | 9 | 78 | 0 | 67 | 67 | 45 |
| SQC | 191 | 40 | 1 | 13 | 18 | 25 |
| Ad-SQ | 45 | 46 | 0 | 22 | 18 | 43 |
| Adeno | 58 | 58 | 0 | 33 | 20 | 20 |
| LCU | 21 | 60 | 0 | 33 | 10 | 33 |
| Total NSCLC | 315 | 44 | 1 | 19 | 9 | 26 |

[a]SQC = Squamous cell carcinoma, Ad-SQ = Mixed adeno-squamous carcinoma, Adeno = Adenocarcinoma, LCU = Large cell undifferentiated carcinoma.

*Expression of markers in tumours from retrospective cases*

Table III summarises the immunohistochemical results. It can be seen that the vast majority of SCLC and carcinoid tumours staining positively for NSE, and that an average of 44% of NSCLC also showed positivity for this marker. Chromogranin A immunostaining was present in one third of carcinoid tumours and in none of the SCLC tumours. Less than 1% of the NSCLC tumours were positive for this marker. CK-BB was detected in 41% and 67% of carcinoid tumours and SCLC tumours respectively. In the NSCLC group CK-BB immunoreactivity was detected on average in 19% of NSCLC tumours. Bombesin like immunoreactivity was present in 30% of carcinoid tumours and in 67% of SCLC. In comparison, 9% of NSCLC were positive for this marker. Neurotensin was detected in 53% of carcinoid tumours, 45% of SCLC and in an average of 26% of NSCLC tumours.

Table IV shows the frequency of tumours expressing nought to four markers. No statistically significant correlation existed between marker positivity and NSCLC histological subtype. It can be seen that 95% of the carcinoid

**Table IV** Percentage of tumours showing 0, 1, 2, 3 and 4 markers retrospective series

| | | *No. of markers* | | | | |
|---|---|---|---|---|---|---|
| | | *0* | *1* | *2* | *3* | *4* |
| *Histology* | *No.* | *% positive* | | | | |
| Carcinoid | 17 | 6 | – | 65 | 18 | 12 |
| SCLC | 9 | 11 | – | 44 | 11 | 33 |
| SQC | 191 | 50 | 24 | 17 | 6 | 3 |
| Ad-SQ | 45 | 31 | 31 | 22 | 9 | 7 |
| Adeno | 58 | 28 | 31 | 20 | 19 | 2 |
| LCU | 21 | 33 | 24 | 19 | 19 | 5 |
| Total NSCLC | 315 | 42 | 28 | 18 | 9 | 3 |

**Table V** Immunohistochemical results of prospective series

| | | *% of tumours showing positivity for* | | | | | | |
|---|---|---|---|---|---|---|---|---|
| *Histology* | *Number* | *NSE* | *CGA* | *CKBB* | *BLP* | *NT* | *Syn* | *UJ-13A* |
| Carcinoid | 3 | 100 | 66 | 66 | 0 | 33 | 66 | 33 |
| SQC | 27 | 50 | 7 | 14 | 4 | 10 | 19 | 14 |
| Ad-SQ | 6 | 100 | 16 | 16 | 16 | 0 | 66 | 16 |
| Adeno | 7 | 42 | 0 | 0 | 14 | 14 | 0 | 14 |
| LCU | 4 | 50 | 0 | 0 | 0 | 0 | 75 | 50 |
| Total NSCLC | 44 | 57 | 7 | 11 | 7 | 10 | 23 | 20 |

tumours and 88% of the SCLC cases showed positivity for two or more markers. Thirty per cent of the NSCLC cases also were positive for two or more of the markers and these were taken to represent NE-NSCLC tumours.

*Expression of markers in tumours from prospective cases*

Table V summarises the immunohistochemical results for the prospective cohort of tumours. No SCLCL cases were treated surgically during the collection time of histological material for this study. Table VI summarises the frequency of neuroendocrine marker positivity according to histologic subtype. As in the retrospective group, it can be seen that 30% of NSCLC tumours showed positivity for two or more markers.

*Ultrastructural features and immunohistochemical results*

Of the 34 cases of NSCLC with material for electron microscopy, 47% showed evidence of DCGs. The number of DCGs varied from case to case with variation in size of granules from 100 to 250 nm. The presence of DCGs correlated significantly with the presence of two or more markers in NE differentiation ($\chi^2 = 5.46$, on 1 d.f., $P = 0.01$) DCGs being detected in nine out of ten NSCLC tumours showing two or more markers (Table VII). All three NSCLC cases which exhibited chromogranin-A positivity in the prospective group showed modest numbers of DCGs. The remaining cases with DCGs did not show immunohistochemical evidence of chromogranin-A positivity.

*Clinical behaviour of NE-NSCLC*

Clinical data for patients in the retrospective group is summarised in Table VIII. The majority of NSCLC patients were staged as T2. A very small number with T3 disease were treated surgically. Similarly, most patients were staged as N-0 and N-1. No N-3 patients were treated surgically. Hence most patients included in the study had stage I and II disease.

**Table VI** Percentage of tumours showing 0, 1, 2, 3, 4 and 5 markers prospective series

| Histology | No. | 0 | 1 | 2 | 3 | 4 | 5 |
|---|---|---|---|---|---|---|---|
| | | No. of markers / No. positive | | | | | |
| Carcinoid | 3 | – | – | – | 1 | 2 | – |
| SQC | 27 | 14 | 5 | 4 | 1 | 3 | – |
| Ad-SQ | 6 | 3 | 1 | 1 | – | – | 1 |
| Adeno | 7 | 2 | 3 | 1 | – | 1 | – |
| LCU | 4 | – | 3 | – | 1 | – | – |
| Total % NSCLC | 44 | 43 | 27 | 14 | 4 | 10 | 2 |

The distribution of NE negative and NE positive NSCLC according to nodal status, disease and tumour stage is shown in Figures 1a–c respectively. It can be seen that the following are NE positive; 26% of N0, 29% of N1 and 44% of N2 cases (Figure 1a); 26% of Stage I, 29% of Stage II, and 43% of Stage III cases (Figure 1b); 26% of T1, 31% of T2 and 43% of T3 cases (Figure 1c). A significant association exists between N-stage and neuroendocrine positivity ($\chi^2 = 6.1$ on 2 d.f., $P = 0.05$) (Figure 1a). The highest proportion of NE positive cases were staged as N2, but a test for trends of increasing NE positivity with N stage was also statistically significant ($\chi^2$ test for linear trend, $\chi^2 = 5.2$ on 1 d.f., $P = 0.02$). Similarly, a significant association exists between overall disease stage and neuroendocrine positivity ($\chi^2 = 6.3$ on 2 d.f., $P = 0.04$) (Figure 1b). A similar trend is apparent for T stage (Figure 1c); however, these data do not reach statistical significance ($\chi^2 = 3.6$ on 2 d.f., $P = 0.17$). Log rank analysis of each of the individual markers was carried out but none showed significant difference in survival between patients positive or negative for the markers. Similarly, there was no difference in survival between patients with and without NE-NSCLC ($\chi^2 = 0.38$ on 1 d.f., $P = 0.54$).

Multivariate analysis of the individual markers, NE status, histology, age, sex, T-stage, N-stage and clinical stage was carried out. The most important prognostic factor was N-

**Table VII** Prospective cases showing positivity for two or more markers

| Case no | Histology | NSE[a] | BLP[a] | NT[a] | Cg-A[a] | CK-BB[a] | UJ1-3A[a] | Synap[a] | NE-I[b] | DCG[c] |
|---|---|---|---|---|---|---|---|---|---|---|
| 4 | Carcinoid | + + + | NS[d] | NS | + + + | + + | NS | + + | 4 | NT[e] |
| 28 | Carcinoid | + + + | NS | NS | NS | + + | NS | + + + | 3 | NT |
| 29 | Carcinoid | + + + | NS | + + | + + + | + | + + | NS | 4 | + + + |
| 3 | SQC | + + + | + + | + + | NS | NS | + + | NS | 4 | + + + |
| 6 | Adeno | + + | + + | + + | NS | NS | + + | NS | 4 | + + + |
| 9 | Adeno-Sq | + + + | NS | NS | + + | + + | + + | + + + | 5 | + + + |
| 11 | SQC | + + | NS | + + | NS | + + | NS | NS | 3 | + |
| 12 | SQC | + + | NS | NS | NS | + + | NS | NS | 2 | – |
| 14 | Adeno-Sq | + + | + + | NS | NS | + | NS | NS | 2 | + + + |
| 17 | SQC | + + + | NS | NS | NS | + + | NS | NS | 2 | + |
| 18 | LCU | + + + | NS | NS | NS | + + | NS | + + + | 3 | + + |
| 25 | SQC | + + + | + | + + | NS | NS | NS | NS | 2 | NT |
| 30 | SQC | + + + | NS | NS | + + + | NS | + + | + + | 4 | NT |
| 38 | SQC | + + | NS | NS | + + | NS | + + | + + | 4 | + + |
| 48 | SQC | + + + | NS | + | NS | + + | NS | NS | 2 | + + |

[a]Intensity of immunostaining; where, + weak; + + moderate; + + + intense staining. [b]NE-I: neuroendocrine index, defined by the presence of 2, 3, 4 or 5 markers. [c]Frequency of DCGs, where: – none; + rare; + + several; + + + many. [d]NS = No staining, [e]NT = No tissue.

**Table VIII** Patient profile of retrospective series

| Histology | No. | Sex: Male | Sex: Female | T stage: T1 | T stage: T2 | T stage: T3 | Nodal stage: N0 | Nodal stage: N1 | Nodal stage: N2 | Disease stage: I | Disease stage: II | Disease stage: III |
|---|---|---|---|---|---|---|---|---|---|---|---|---|
| Carcinoid[a] | 17 | 9 | 8 | 10 | 1 | – | 8 | 3 | – | 8 | 3 | – |
| SCLC | 9 | 5 | 4 | 3 | 6 | – | 3 | 6 | – | 3 | 6 | – |
| SQ.CC | 191 | 157 | 34 | 65 | 117 | 9 | 116 | 50 | 25 | 110 | 49 | 32 |
| Ad-SQ | 45 | 39 | 6 | 7 | 35 | 3 | 23 | 12 | 10 | 23 | 10 | 12 |
| Adeno | 58 | 43 | 15 | 19 | 38 | 1 | 35 | 12 | 11 | 34 | 12 | 12 |
| LCU | 21 | 17 | 4 | 6 | 14 | 1 | 11 | 4 | 6 | 10 | 4 | 7 |
| | 315 | 256 | 59 | 97 | 204 | 14 | 185 | 78 | 52 | 177 | 75 | 63 |

Total NSCLC. [a]Precise TNM data were not available for all tumours.

**Figure 1a** NE status and nodal stage in NSCLC tumours. ▒, N0 ($n = 185$); ▨, N1 ($n = 78$); ■, N2 ($n = 52$). **b**, NE status and disease stage in NSCLC tumours. ▒, Stage I ($n = 177$); ▨, Stage II ($n = 75$); ■, Stage III ($n = 63$). **c**, NE status and T stage of NSCLC tumours. ▒, T1 ($n = 97$); ▨, T2 ($n = 204$); ■, T3 ($n = 14$).

stage ($\chi^2 = 36.2$ on 1 d.f., $P < 0.0001$), followed by T-stage ($\chi^2 = 11.4$ on 1 d.f., $P = 0.0007$) but no other factor added to these two (at a significance level of less than 0.05).

### Discussion

The observed specificity for normal NE tissues shown by the markers selected to identify NE-NSCLC tumours suggests that the presence of one or more NE markers in NSCLC tumours reflects varying degrees of NE differentiation in these tumours.

In the present study approximately 50% of NSCLC tumours stained positively for NSE. Similar findings have been reported by others (Bergh *et al.*, 1985; Said *et al.*, 1985; Rode *et al.*, 1985; Dhillon *et al.*, 1985; Linnoila *et al.*, 1988*b*; Graziano *et al.*, 1989). The detection of CK-BB immunoreactivity in 67% of SCLC and 14% of squamous cell tumours compares well with previous radioimmunoassay data (Ruberry *et al.*, 1983). While several studies have failed to detect bombesin like immunoreactivity in NSCLC using immunohistochemistry (Addis *et al.*, 1987; Hamid *et al.*, 1987), a recent study by Linnoila *et al.*, 1988*b*, reported the immunohistochemical detection of bombesin like immunoreactivity in 13% of NSCLC. In the present study, 8% of NSCLC tumours showed bombesin like immunoreactivity. The presence of neurotensin in adenocarcinomas has also been previously described (Dammrich *et al.*, 1988). The additional detection of neurotensin in squamous cell and large cell undifferentiated carcinomas in the present study, possibly reflects the larger numbers examined. NCAM positivity was observed in frozen tumour material in one of three carcinoid tumours and in 20% of non-SCLC cases. A study by Gatter and Dunnill, 1985, also demonstrated the expression of NCAM by carcinoid tumours, and, these workers described focal positivity for this molecule in some NSCLC tumours. Similarly, Moss *et al.*, 1986, have also reported UJ-13A immuno-reactivity in large cell lung carcinomas. Synaptophysin was detected in approximately 23% of NSCLC cases. In view of its restricted use in acetyl alcohol fixed tissues, the role of synaptophysin in diagnostic pathology is limited.

An important feature of the present study is the correlation between the presence of DCGs and the detection of two or more biochemical NE markers in the prospective group of NSCLC. Although DCGs were identified by electron microscopy, few NSCLC showed evidence of immunoreactivity for chromogranin-A. Similar findings have been reported by others (Linnoila *et al.*, 1988*b*; Grazianno *et al.*, 1989). Such findings may indicate that NSCLC tumours do not have sufficient DCGs to enable detection by immunocytochemistry.

Since cell differentiation is characterised by the coordinated expression of cell specific groups of genes, the detection of multiple NE markers within a single neoplasm is likely to delineate NSCLC tumours with a true endocrine profile. In the present study the expression of two or more markers identified all but one of the carcinoid tumours and all SCLCs and, therefore, was taken to define the neuroendocrine phenotype. Using this criterion, 30% of NSCLC tumours in both the retrospective and prospective group were found to be neuroendocrine. These results compare well with those reported previously (Linnoila *et al.*, 1988*b*; Graziano *et al.*, 1989; Berendsen *et al.*, 1989). Importantly, tumours positive for two or more markers in our consecutive surgical series were associated with nodal involvement, particularly N2 disease. Furthermore, such tumours were associated with an increased likelihood of stage II disease. These findings indicate that NE-NSCLC tumours appear to be more frequently highly metastatic than non-NE tumours and supports the contention, based on chemosensitivity studies, that the clinical behaviour of these tumours is similar to that of SCLC. The findings of this study also show that NE differentiation in NSCLC is of no prognostic significance with respect to survival. The ongoing prospective study initiated in parallel with this retrospective investigation will determine further whether the presence of NE markers identifies disease with an inherently more aggressive natural history than that of non-NE-NSCLC.

The authors wish to thank Mrs Beverly Wilson (senior MLSO), Dr D.G.D. Wight (Consultant Histopathologist), Department of Histopathology, Addenbrooke's Hospital, Cambridge, Mr F.C. Wells (Consultant Cardiothoracic Surgeon), Papworth Hospital, Mr R.J. Kingshott (Senior Chief MLSO), Department of Histopathology, Papworth Hospital, Cambridge, Mr Terry Bull (EM MLSO), and Dr B. Fox (Consultant Histopathologist), Department of Histopathology, Charing Cross Hospital, London, UK.

This work was funded by the Cancer Research Campaign.

### References

ADDIS, B.J, HAMID, Q., IBRAHIM, N.B.N., FAHEY, M., BLOOM, S.R. & POLAK, J.M. (1987). Immunohistochemical markers of small cell carcinoma and related neuroendocrine tumours of the lung. *J. Pathol.*, **153**, 137.

ALLAN, P.M., GARSON, J.A., HARPER, E.I. & 4 others (1983). Biological characterisation and clinical application of a monoclonal antibody recognising an antigen restricted to neuroectodermal tissue. *Int. J. Cancer*, **31**, 591.

BAYLIN, S.B., ABELOFF, M.D., GOODWIN, G., CARNEY, D.N. & GAZDAR, A.F. (1980). Activities of L-dopa decarboxylase and diamine oxidase (histaminase) in lung cancers and decarboxylase as a marker for small (oat) cell cancer in culture. *Cancer Res.*, **40**, 1990.

BERENDSEN, H.H., DE LEJI, L., POPPEMA, S., POSTMUS, P.E., BOES, A., SLUITER, H.J. & THE, H. (1989). The clinical characterization of non-small cell lung cancer tumours showing neuroendocrine differentiation features. *J. Clin. Oncol.*, **7**, 1614.

BERGER, C.L., GOODWIN, G., MENDELSOHN, G. & 4 others (1981). Endocrine related biochemistry in the spectrum of human lung carcinoma. *J. Clin. Endocrin. & Metab.*, **52**, 422.

BERGH, J., ESSCHER, T., STEINHOLTZ, L., NILSSON, K. & PAOHLMAN, K. (1985). Immunocytochemical demonstration of neuron specific enolase (NSE) in human lung cancers. *Am. J. Clin. Pathol.*, **84**, 1.

BISHOP, A.E., POWER, R.F. & POLAK, J.M. (1988). Markers for neuroendocrine differentiation. *Path. Res. Pract.*, **183**, 119.

DAMMRICH, J., ORMANNS, W., KAHALY, G. & SCHREZENMEIR, J. (1988). Multiple peptide producing adenocarcinoma of lung with neurotensin and CRF - like immunoreactivity. *Path. Res. Pract.*, **183**, 670.

DHILLON, A.P., RODE, J., DHILLON, D.P. & 4 others (1985). Neural markers in carcinoma of the lung. *Br. J. Cancer*, **51**, 645.

GATTER, K.C., DUNNILL, M.S., PULFORD, K.A.F., HERYET, A. & MASON, D.Y. (1985). Human lung tumours: a correlation of antigenic profile with histologic type. *Histopath.*, **9**, 805.

GAZDAR, A.F., ZWEIG, M.H., CARNEY, D.N., VAN STEIRTEGHEN, A.C., BAYLIN, S.B. & MINNA, J.D. (1981). Levels of creatinine kinase and its BB isoenzyme in lung cancer specimens and cultures. *Cancer Res.*, **41**, 2773.

GAZDAR, A.F. (1986). Advances in the biology of non-small cell lung cancer. *Chest*, **89**, 277S.

GAZDAR, A.F., HELMAN, L.J., ISRAEL, M.A. & 5 others (1988*a*). Expression of neuroendocrine cell markers L-dopa decaboxylase, chromogranin A, and dense core granules in human tumours of endocrine and nonendocrine origin. *Cancer Res.*, **48**, 4078.

GAZDAR, A.F., TSAI, C.M. & PARK, J.G. (1988*b*). Relative chemosensitivity of non-small cell lung cancers expressing neuroendocrine properties (Abs). *Proc. Am. Soc. Clin. Oncol.*, **7**, 200.

GOULD, V.E., LEE, I., WIEDMAN, B., MOLL, R., CHEJFEC, G. & FRANKE, W.W. (1986). Synaptophysin: a novel marker for neurons, certain neuroendocrine cells and their neoplasms. *Hum. Path.*, **17**, 979.

GRAZIANO, S.L., MAZID, R., NEWMAN, N. & 6 others (1989). The use of neuroendocrine immunoperoxidase markers to predict chemotherapy response in patients with non-small cell lung cancer. *J. Clin. Oncol.*, **17**, 1398.

HAMID, Q.A., ADDIS, B.J., SPRINGALL, D.R. & 4 others (1987). Expression of the C-terminal peptide of human pro-bombesin in 361 lung endocrine tumours, a reliable marker and possible prognostic indicator for small cell carcinoma. *Virch. Arch. A.*, **411**, 185.

HERMANEK, P. & SOBIN, L.H. (1987). *TNM Classification of Malignant Tumours*. Springer-Verlag: London.

LINNOILA, R.I., JENSEN, S., STEINBERG, S., MINNA, J., GAZDAR, A.F. & MULSHINE, J.L. (1988*a*). Neuroendocrine differentiation correlates with favourable response to chemotherapy in patients with non-small cell lung cancer. *Lung Cancer*, **4**, A33 (Abstr).

LINNOILA, R.I., MULSHINE, J.L., STEINBERG, S.M. & 4 others (1988*b*). Neuroendocrine differentiation in endocrine and nonendocrine lung carcinoma. *Am. J. Clin. Path.*, **90**, 641.

MARANGOS, P.J., GAZDAR, A.F. & CARNEY, D.F. (1982). Neuron-specific enolase in human small cell carcinoma cultures. *Cancer Lett.*, **15**, 7.

MOODY, T.W., PERT, C.B., GAZDAR, A.F., CARNEY, D.D. & MINNA, J.D. (1981). High levels of intracellular bombesin characterise human small cell lung carcinoma. *Science*, **214**, 1246.

MOSS, F., BOBROW, L.G., SHEPPARD, M.N. & 5 others (1986). Expression of epithelial and neural antigens and small cell and non small cell lung carcinoma. *J. Pathol.*, **149**, 103.

MULSHINE, J., IHDE, D., LINNOILA, R.I., VEACH, S. & 5 others (1987). Preliminary report of a prospective trial of neuroendocrine marker analysis and in vitro drug sensitivity testing in patients with non-small cell lung cancer. *Proc. Am. Soc. Clin. Oncol.*, **6**, 713, (Abst.).

PATEL, K., ROSSELL, R.J., BOURNE, S., MOORE, S.E., WALSH, F.S. & KEMSHED, J.T. (1989). Monoclonal antibody UJ13A recognises the neural cell adhesion molecule (NCAM). *Int. J. Cancer*, **44**, 1062.

PETO, R., PIKE, M.C., ARMITAGE, P. & 7 others (1977). Design and analysis of randomised clinical trials requiring prolonged observation of each patient. *Br. J. Cancer*, **35**, 1.

RODE, J., DHILLON, J.F., DORAN, J.F., JACKSON, P. & THOMPSON, R.J. (1985). PGP 9.5, a new marker for human neuroendocrine tumours. *Histopath.*, **9**, 147.

RUBERRY, R.D., DORAN, J.F. & THOMPSON, R.J. (1983). Brain type creatine kinase BB as a potential tumour marker - serum levels measured by radioimmunoassay in 1015 patients with histologically confirmed malignancies. *Eur. J. Cancer Clin. Oncol.*, **18**, 951.

SAID, J.W., VIMALADAL, S., NASH, G. & 4 others (1985). Immunoreactive neuron specific enolase, bombesin and chromogranin as markers for neuroendocrine lung tumours. *Human Path.*, **16**, 236.

SCHMECHEL, D.E., MARANGOS, P.J. & BRIGHTMAN, M.M. (1978). Neuron-specific enolase is a molecular marker for peripheral and central neuroendocrine cells. *Nature*, **276**, 834.

TIBSHIRANI, R. (1982). A plain man's guide to the proportional hazards model. *Clin. Invest. Med.*, **5**, 63–68.

WORLD HEALTH ORGANISATION (1981). Histological typing of lung tumours. *2nd ed. International Classification of Tumours*. No. 1. WHO Geneva.

WILKINSON, K.D., LEE, K., DESHPANDE, S., DUERKSEN-HUGHES, P. & BOSS, J.M. (1989). The neuron specific protein PGP 9.5 is a ubiquitin caboxyl-terminal hydrolase. *Science*, **246**, 670.

WILSON, P.O.G., BARBER, P.C., HAMID, Q.A. & 6 others (1988). The immunolocalization of protein gene product 9.5 using rabbit polyclonal and mouse monoclonal antibodies. *Br. J. Exp. Path.*, **69**, 91.

YAMAGUCHI, K., ABE, K., ADACHI, I. & 6 others (1985). Peptide hormone production in primary lung tumours. *Recent Results in Cancer Res.*, **99**, 107.